# Supplementary figures and images for: The alignment model of indirect communication
Source: PLoS One. 2025 May 28;20(5):e0323839. doi: 10.1371/journal.pone.0323839 (PMC12118985; doi:10.1371/journal.pone.0323839)

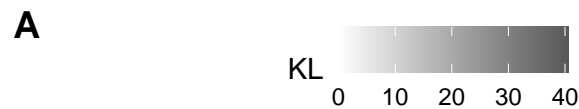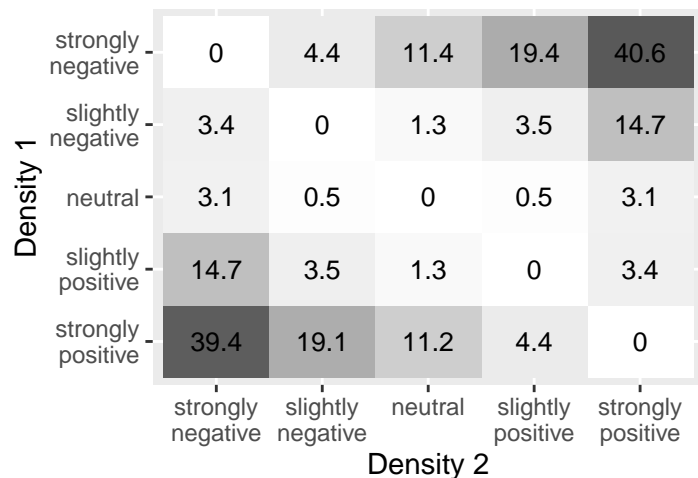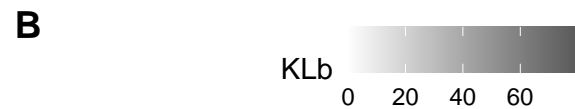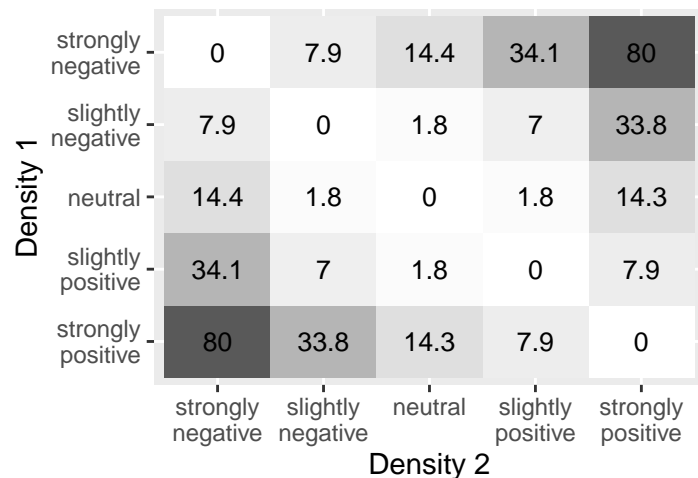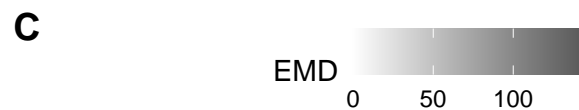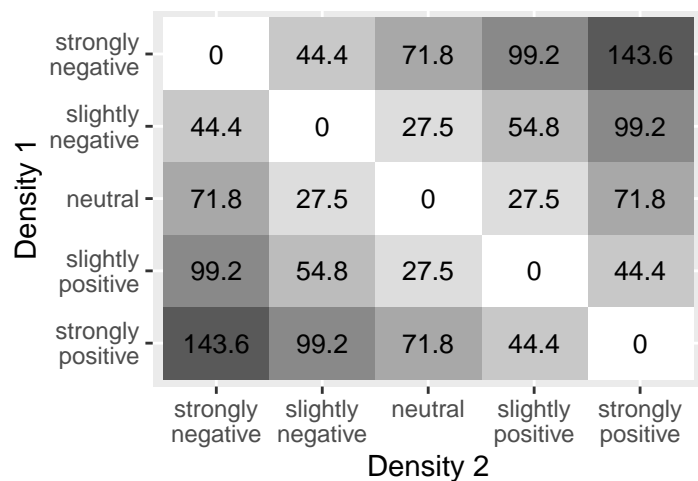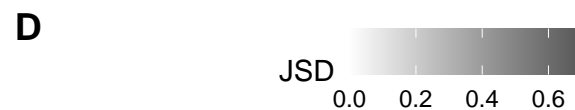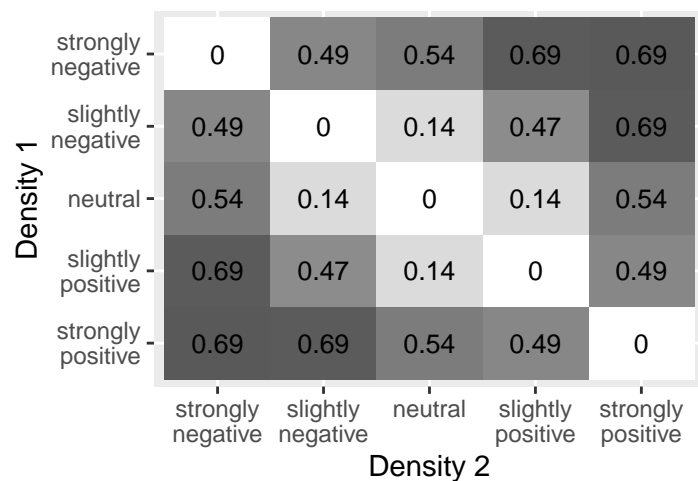

Supplement: S1 Fig — Measures of divergence between the opinion distributions may be interpreted as encoding the effort to change one belief into another one, or, in other words, belief compatibility. a) Kullback Leibler (KL) divergence; b) Bidirectional KL-divergence; c) Earth Mover’s Distance; d) Jensen-Shannon Divergence (PDF) [file pone.0323839.s001.pdf]

## How may the second speaker actually feel about the issue?

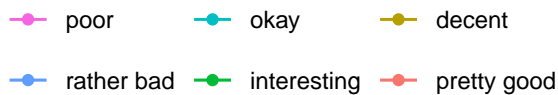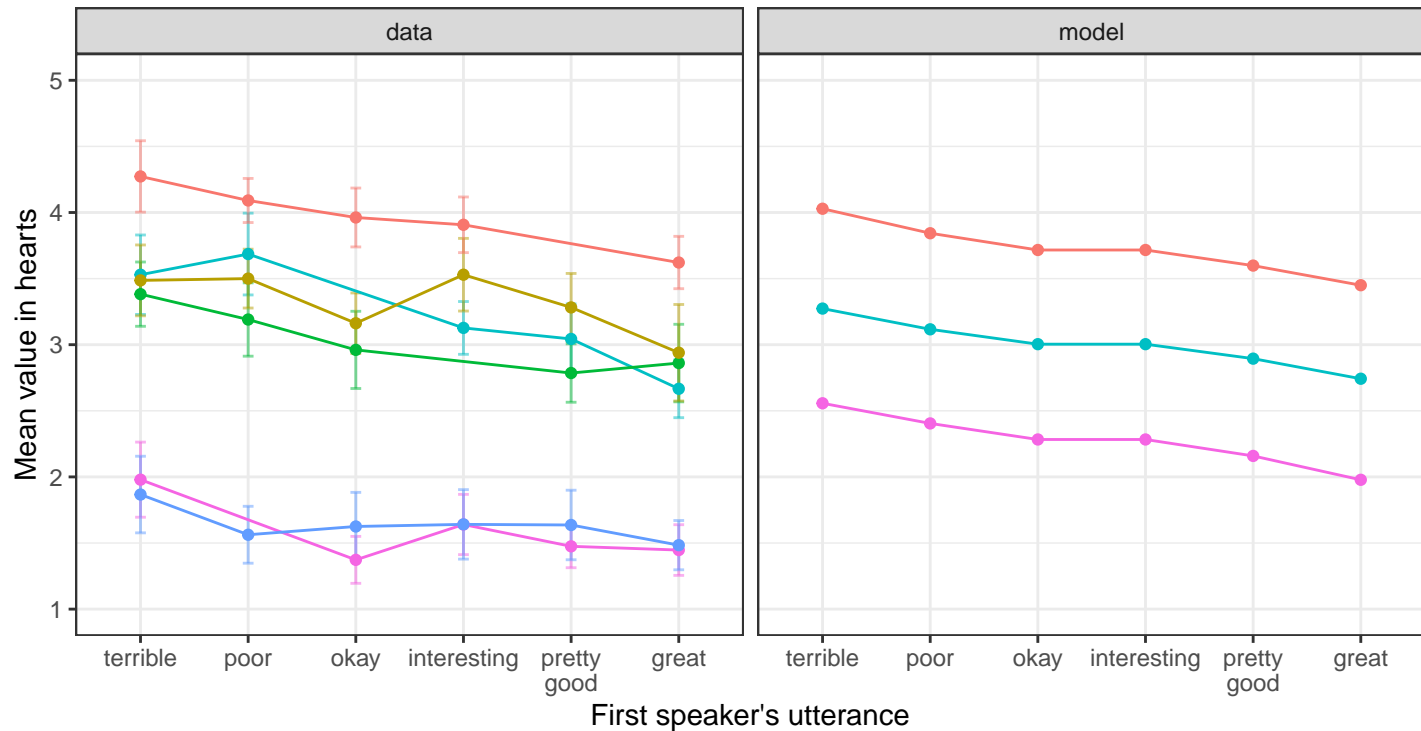

Supplement: S2 Fig — Unidirectional KL-divergence produces a similar qualitative pattern compared to the bidirectional KL-divergence: the model infers a more positive opinion of speaker B given a more negative utterance of speaker A. (PDF) [file pone.0323839.s002.pdf]

## How may the second speaker actually feel about the issue?

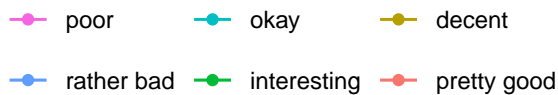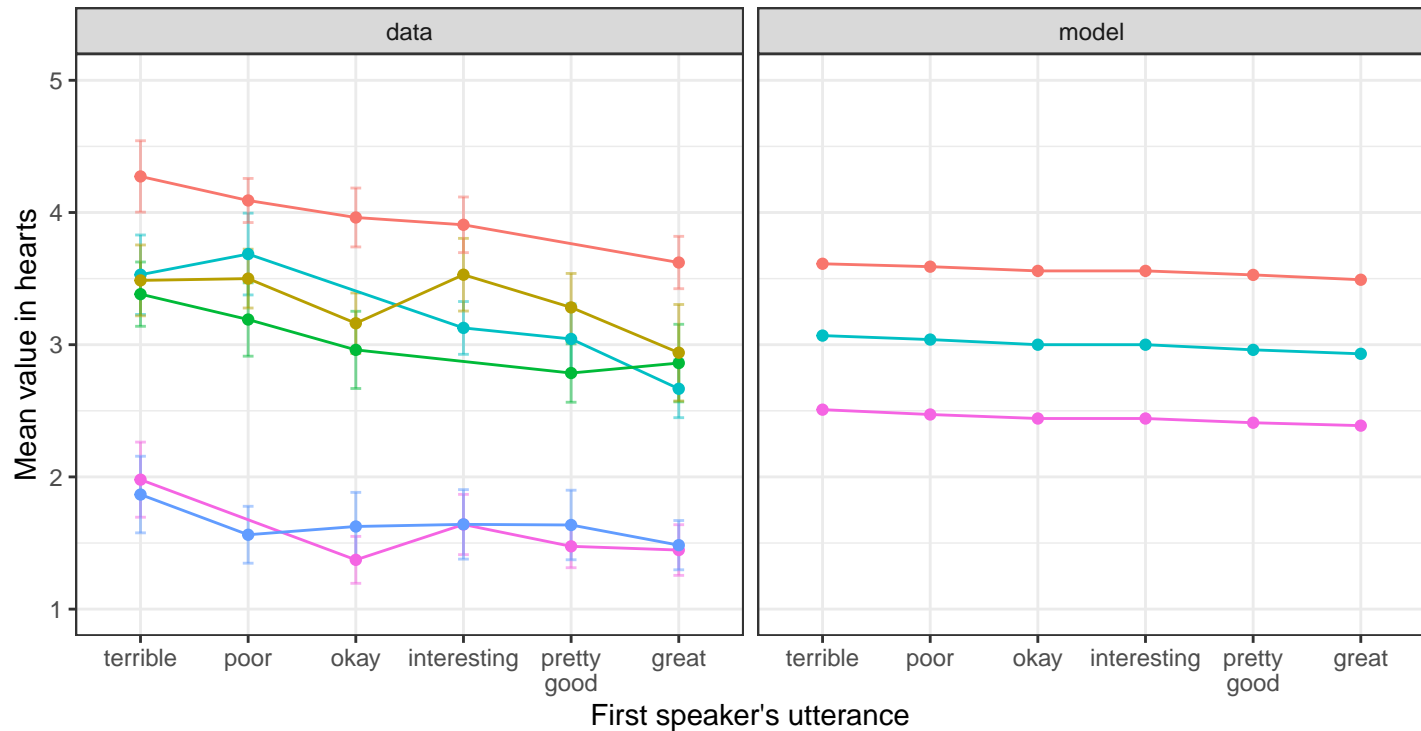

Supplement: S3 Fig — Jensen-Shannon Divergence (Fig (15)) shows a weaker relationship between the first speaker’s utterance and the inferred opinion. (PDF) [file pone.0323839.s003.pdf]

## How may the second speaker actually feel about the issue?

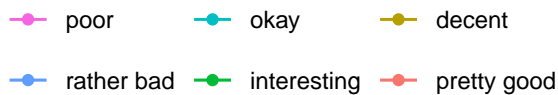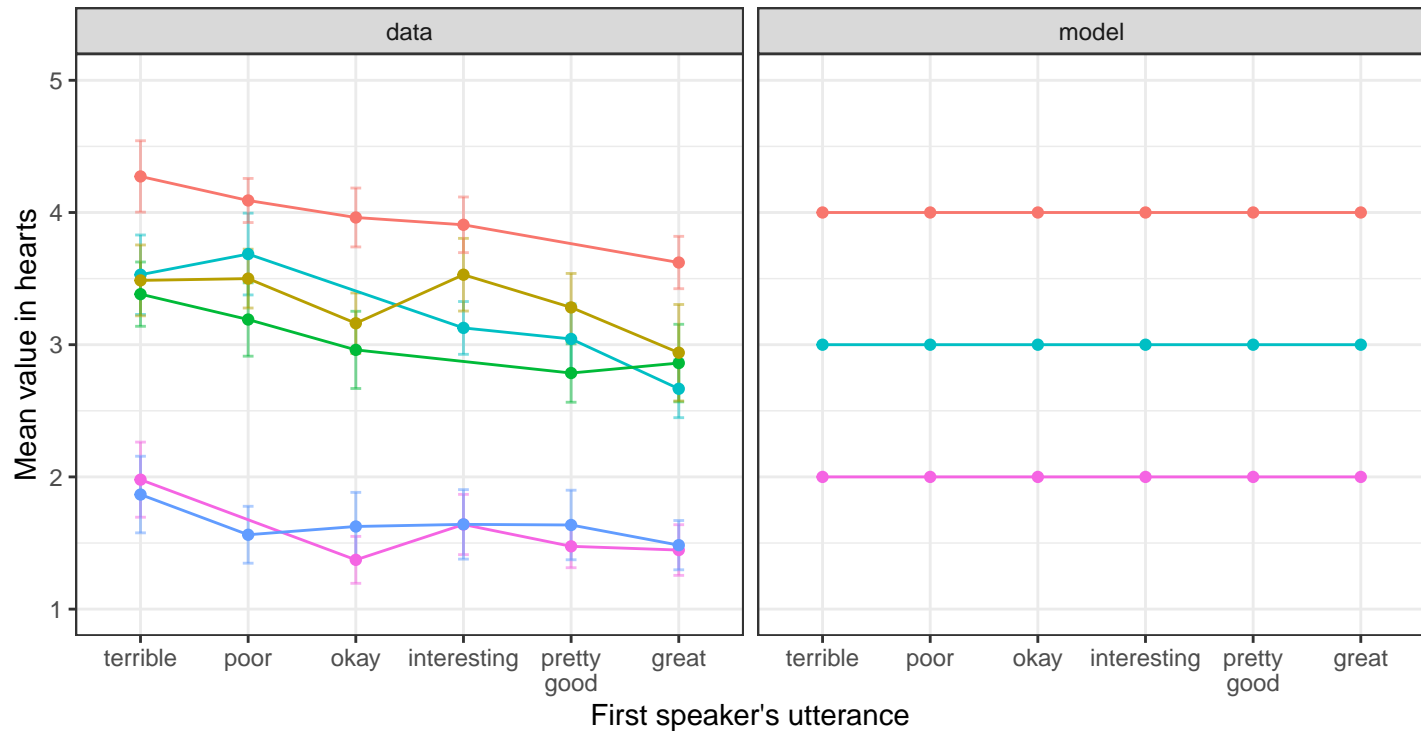

Supplement: S4 Fig — The Earth Mover’s Distance (Fig (15)) even with modified parameters fails to capture the qualitative pattern observed in the data. (PDF) [file pone.0323839.s004.pdf]

# How may the second speaker actually feel about the issue?

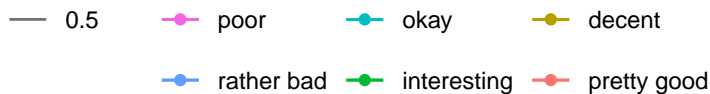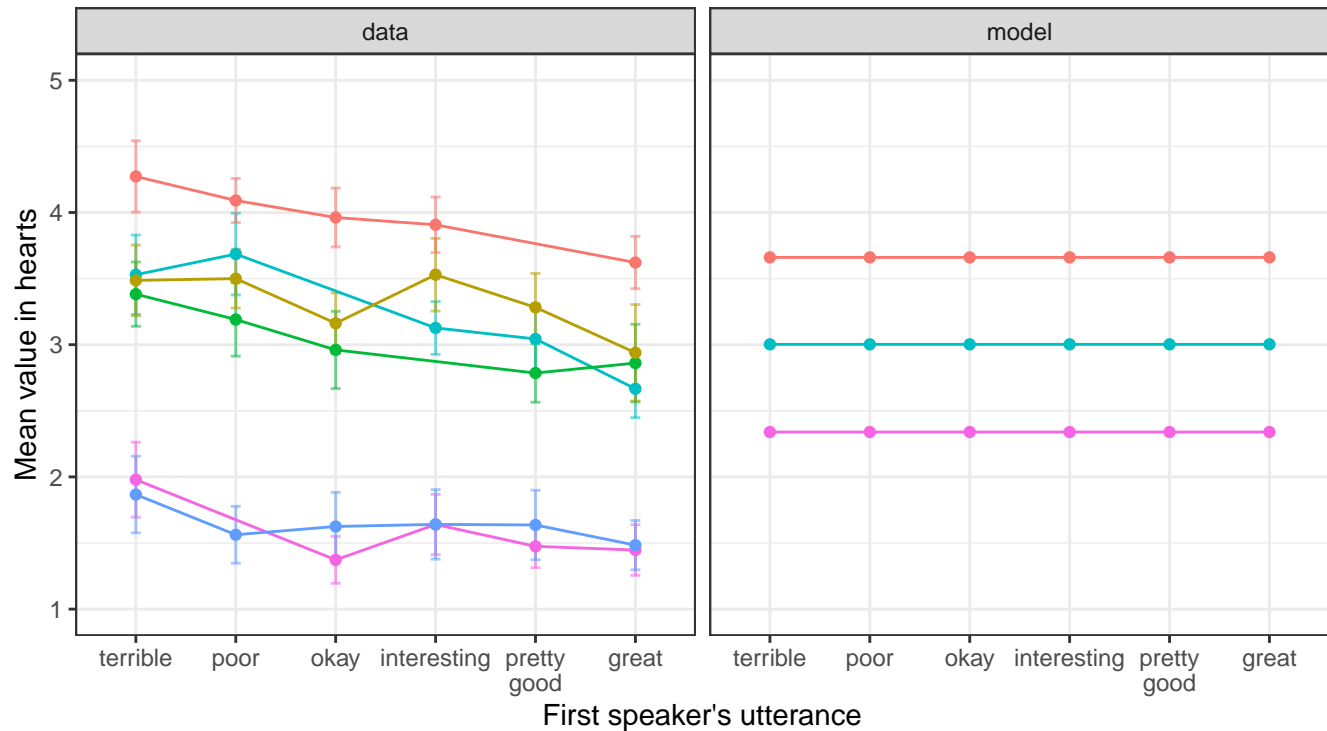

Supplement: S5 Fig — The inferred second speaker’s opinion does not depend on the utterance of the first speaker: the model infers that the speaker says what she means, since the social goal has been set to 0. (PDF) [file pone.0323839.s005.pdf]

**A**

Utterance utility

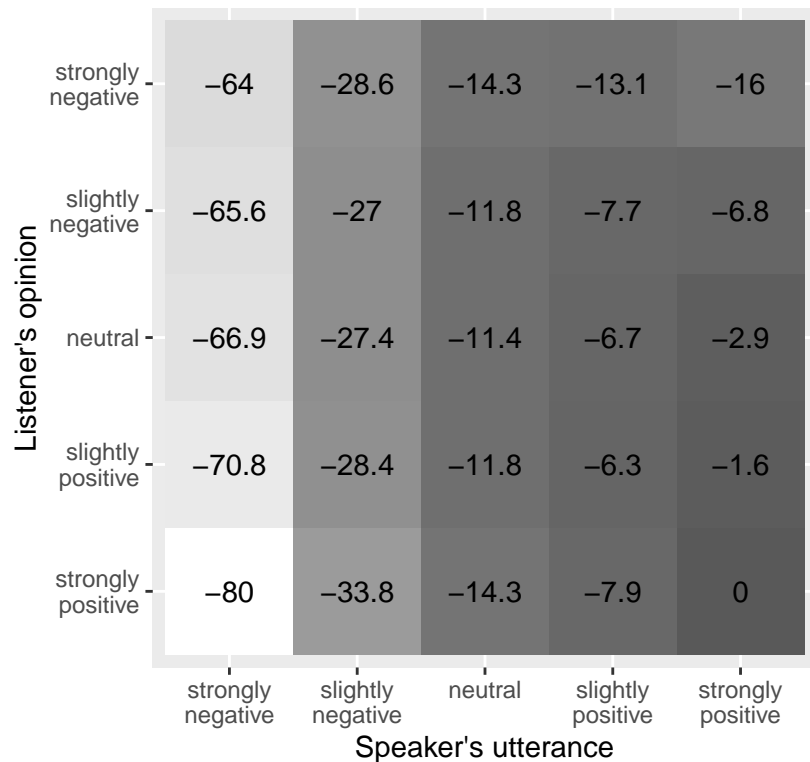**B**

Utterance choice probability

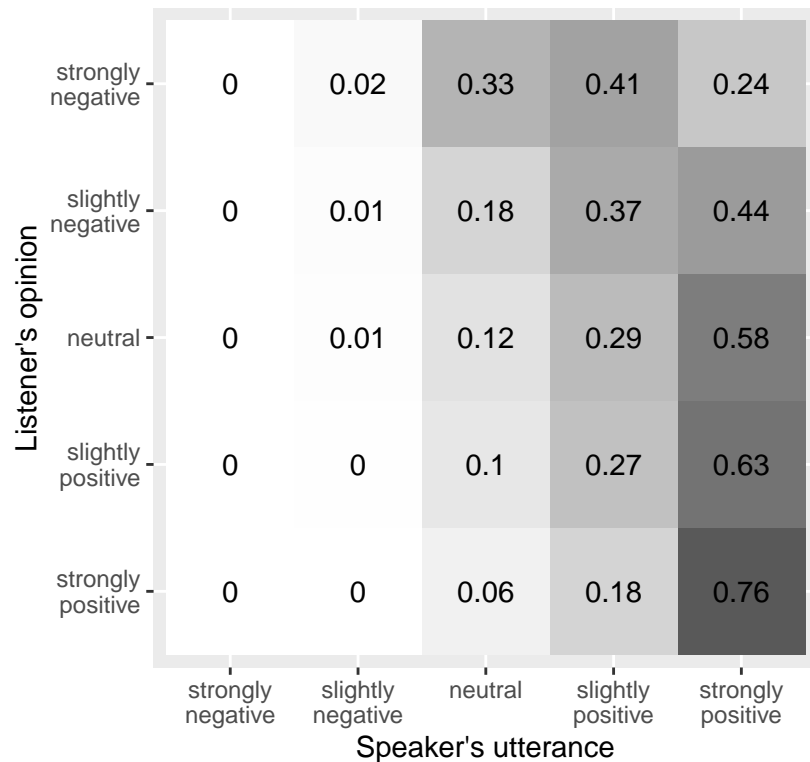

Supplement: S6 Fig — The calculations assume that the speaker’s opinion corresponds to a strongly positive (α=30,β=5) opinion distribution. Panel A shows utility values for utterances (rows) given different speaker beliefs about the listener’s opinion state π1S1 (here assumed to be single-peaked distributions). Panel B shows the corresponding utterance-choice probabilities computed via Equation (7) assuming ωinf=0.8, ωsoc=0.2, and α=0.18. The values show that the model generates progressively smaller utilities for utterances that diverge from the speaker’s opinion (strongly positive, in this case). Generally, utterances that offer the best compromise between the speaker’s opinion and the believed listener’s opinion are preferred. (PDF) [file pone.0323839.s006.pdf]

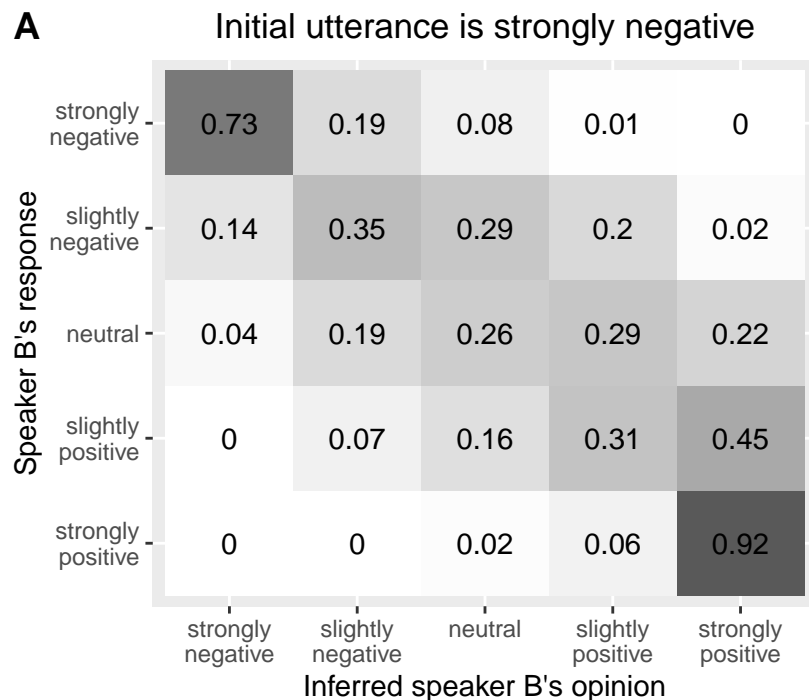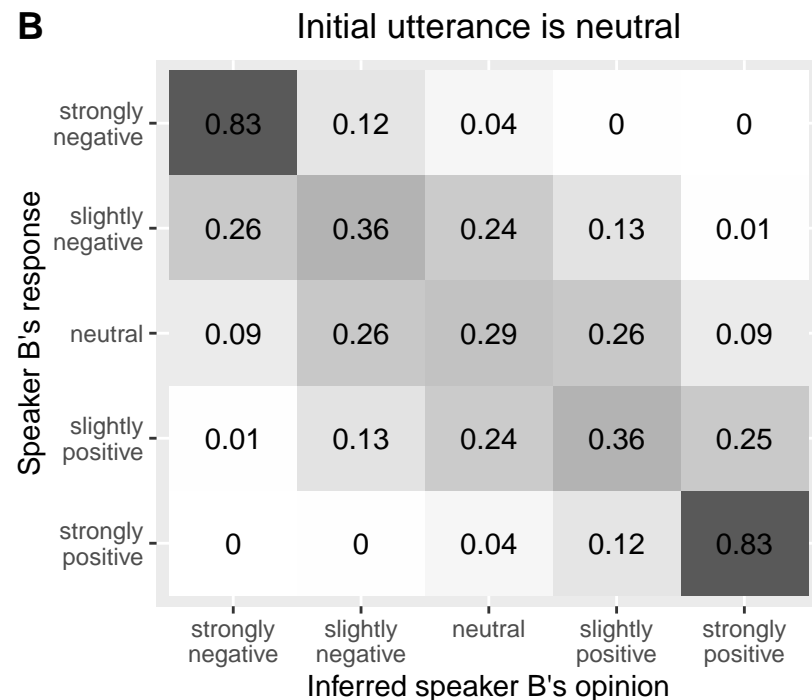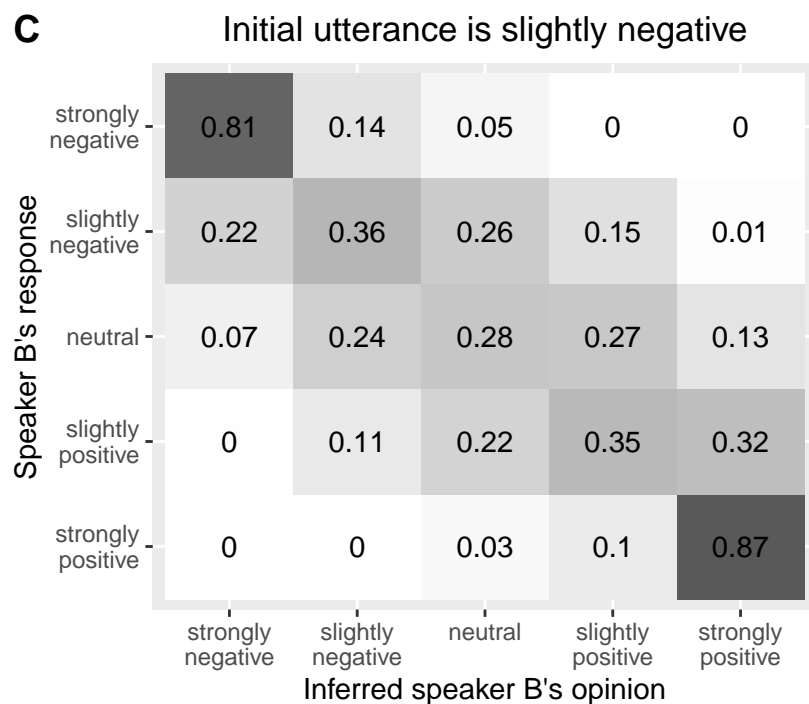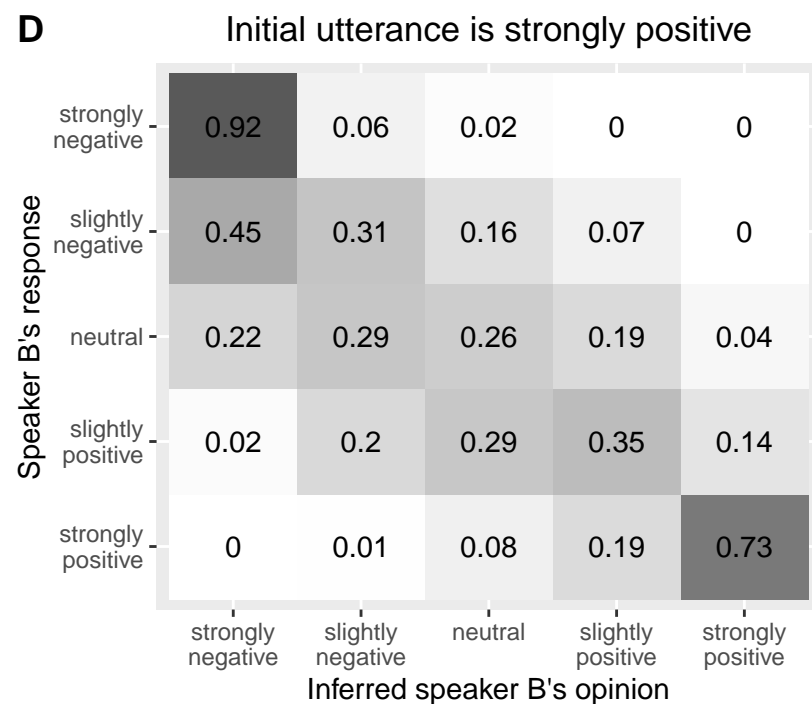

Supplement: S7 Fig — Model’s posterior estimation of speaker B’s opinion computed via Equation (8) given an initial utterance that is strongly negative (panel A), strongly positive (panel B), neutral (panel C), or slightly positive (panel D). Each row in each matrix encodes a particular posterior belief distribution π1A over speaker B’s opinion given her response indicated in each row. (PDF) [file pone.0323839.s007.pdf]

Proportion of responses

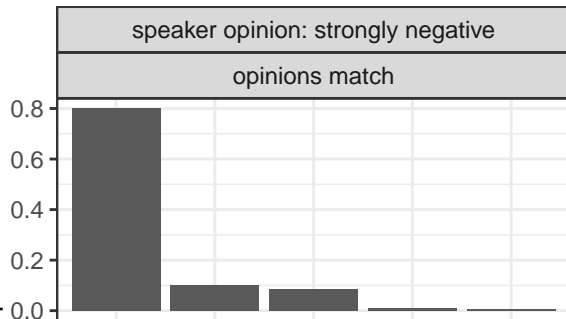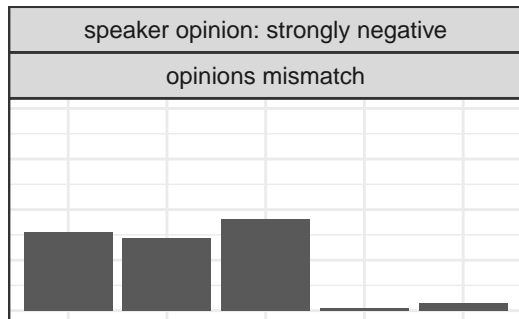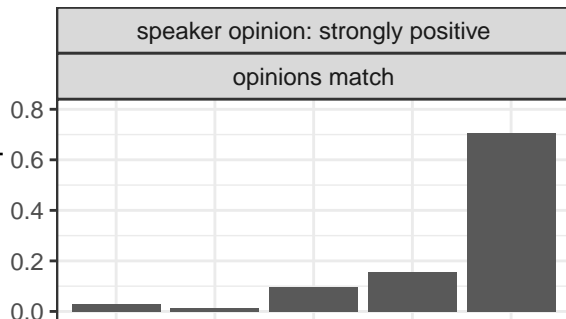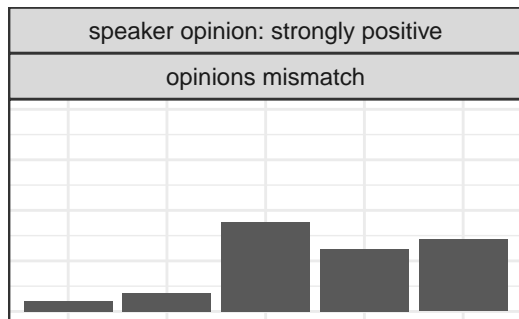

Utterance

Supplement: S8 Fig — The left column shows the cases were the opinions of conversation partners match. Here speakers prefer utterances that correspond to their true opinion. The right column shows the cases of mismatch in opinion. Utterance choices shift towards the middle of the scale. (PDF) [file pone.0323839.s008.pdf]

Proportion of responses

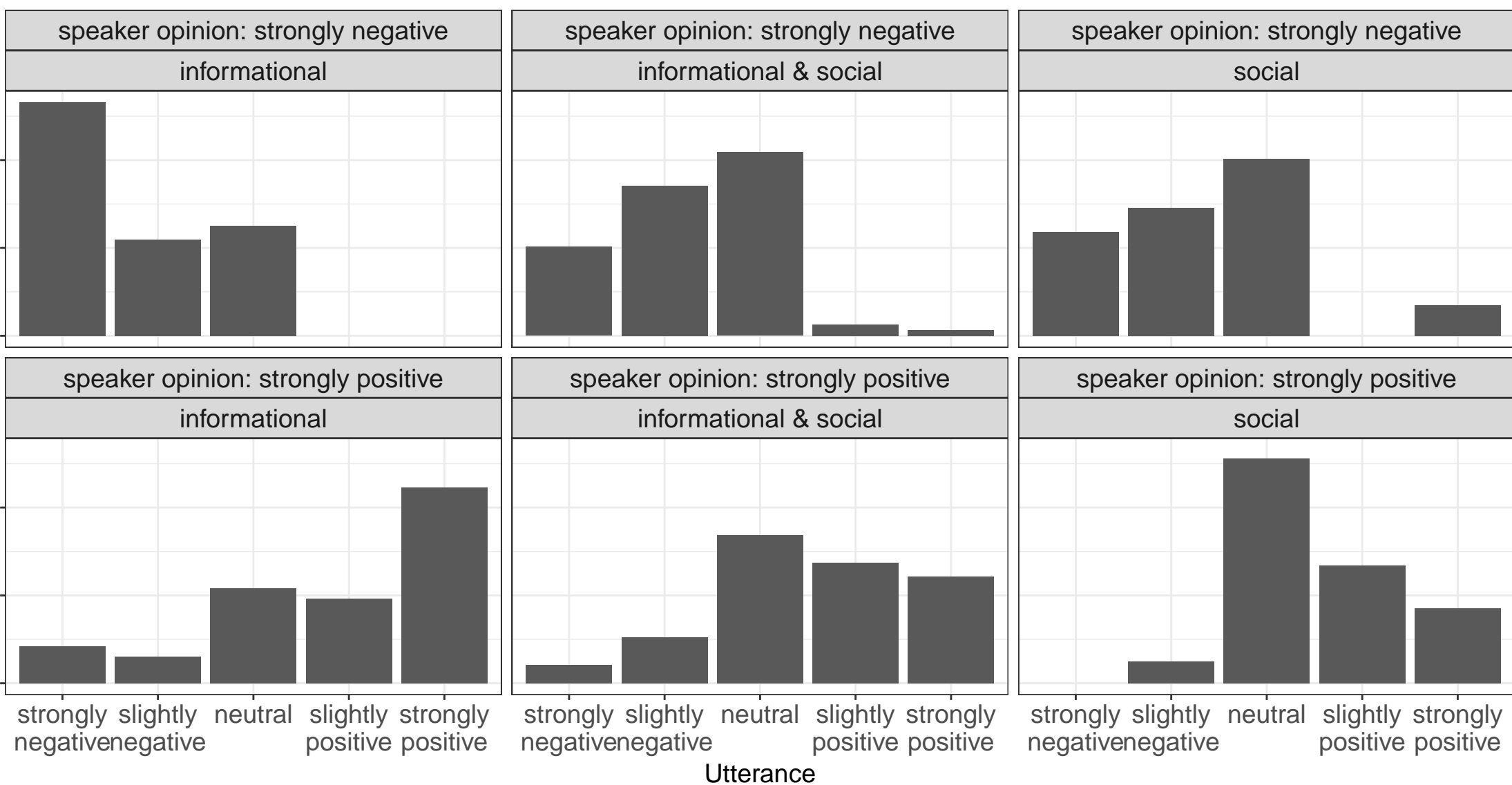

Supplement: S9 Fig — Cases where the opinions of conversation partners do not match. The top row corresponds to the strongly negative opinion of the speaker, the bottom row shows the strongly positive opinion of the speaker. (PDF) [file pone.0323839.s009.pdf]

# How may the second speaker actually feel about the topic?

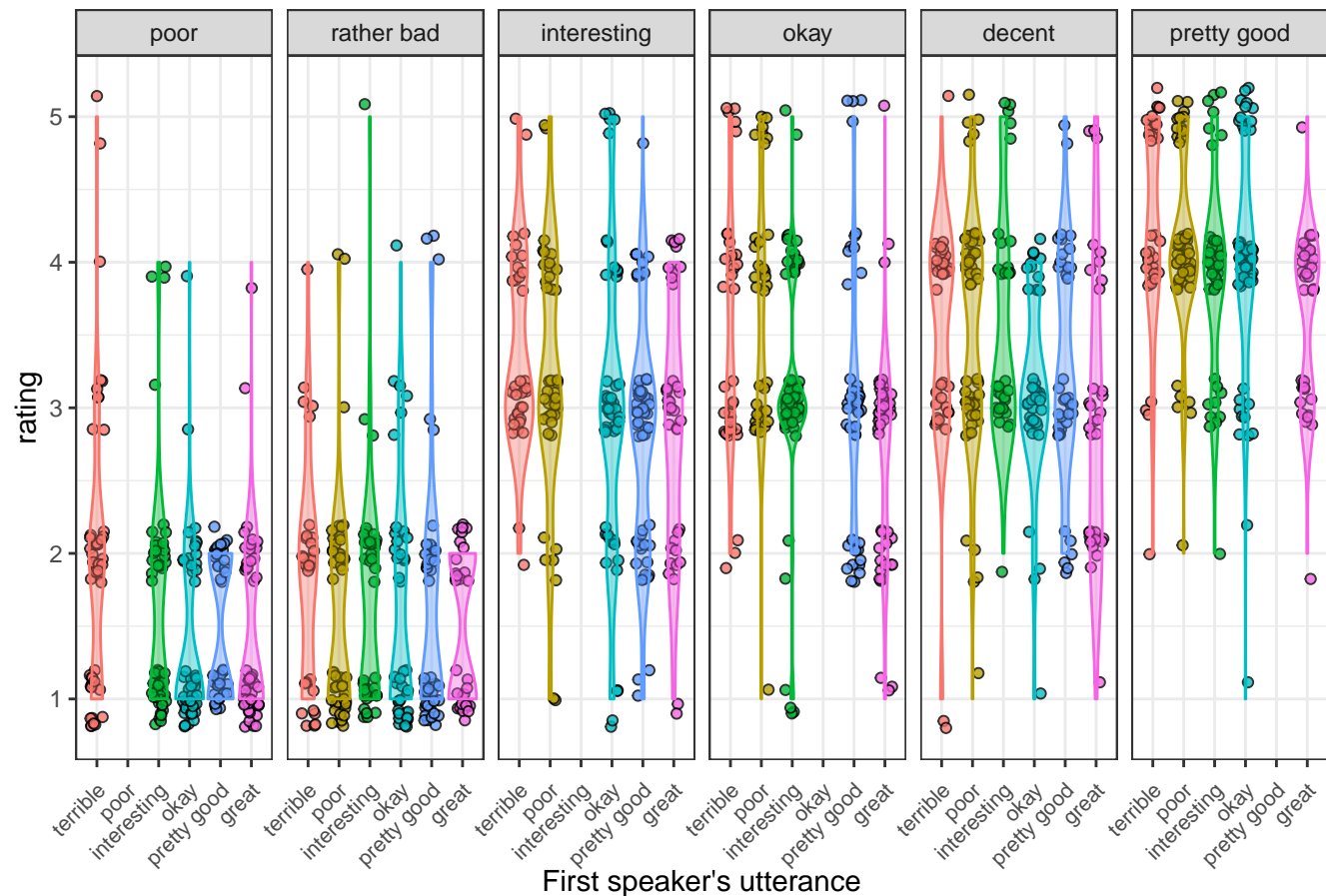

Supplement: S10 Fig — Each data point represents a participant’s response. Jitter was added for visualization purposes. The location of the clusters along the vertical axis reflects how positive the inferred opinion is. Thus, the relevant contrasts lie within each facet between the adjectives at the opposite ends of the scale. The model predicts that upon hearing a predicate, such as “interesting”, participants should infer the opinion as more positive if the predicate follows a strongly negative statement compared to a strongly positive statement. (PDF) [file pone.0323839.s010.pdf]
