# Supplementary material for: The alignment model of indirect communication
Source: PLoS One. 2025 May 28;20(5):e0323839. doi: 10.1371/journal.pone.0323839 (PMC12118985; doi:10.1371/journal.pone.0323839)
Supplement: Attachment — Submitted filename: Achimova_Franke_Butz_Revisions_letter.pdf [file pone.0323839.s011.pdf]

EBERHARD KARLS
UNIVERSITÄT
TÜBINGEN

Faculty of
Humanities

Department of Linguistics

Dr. Asya Achimova

Phone +49 7071 29-73953
asya.achimova@uni-tuebingen.de

Tübingen, December 11, 2024

Dear editors,

We have revised our submission PONE-D-24-54951 and made the following correction:

We have previously attached the wrong ethics decision letter to the submission. The correct ethics decision letter is now attached, it covers the period of testing for the experiments that we report. We apologize for our error and hope you can consider the new submission.

Sincerely yours,

Asya Achimova, Michael Franke, and Martin V. Butz
