# Supplementary material for: The alignment model of indirect communication
Source: PLoS One. 2025 May 28;20(5):e0323839. doi: 10.1371/journal.pone.0323839 (PMC12118985; doi:10.1371/journal.pone.0323839)
Supplement: Attachment — Submitted filename: achimova_franke_butz_revisions_letter_auresp_1.pdf [file pone.0323839.s012.pdf]

Response to reviewers

Asya Achimova, Michael Franke, Martin V. Butz

April 4, 2025

Dear editors, dear reviewers,

Thank you for your assessment and feedback on the previous version of our paper! Your suggestions helped us sharpen the concepts of indirect communication and contextualize our paper better in the existing literature. In the letter below, we detail how we address each of the concerns you have raised.

Reviewer 1

Review 1

1. This is a very good article that deserves publication, pending some revisions regarding, in particular, the distinction between vagueness/indirectness and the methodological section, where important information is missing.

   We appreciate Reviewer 1's assessment of our manuscript and hope that we were able to address the concerns that he raised.

2. Introduction: the authors' rationale could also be connected with the idea that the meaning of an evaluative statement is assessed against other possible alternatives, as in the case of negative statements (e.g., not overtly bright is assessed against not bright, stupid, etc.), which are also mentioned later on.

   We now more explicitly mention that indirect utterances are evaluated against alternatives and cite relevant literature in the *Background* section.

3. L73: You could provide some references to indirectness, which is defined in somewhat vague terms in the current state of the manuscript.E.g., Ruytenbeek, N. (2024). Indirectness. In Handbook of Pragmatics: 27th Annual Installment (pp. 101-127). John Benjamins Publishing Company. (for an overview of different notions of indirectness and a critical discussion).

   We are grateful for the suggested reference on indirect communication! We have now incorporated a more substantial discussion of indirect communication into the *Background* section.

4. L85: it is unclear if this has to do with vagueness or indirect communication; in the philosophy of language, vagueness is a distinct concept, and it is more specific to lexical items than to constructions or utterances. On vagueness, see e.g., Égré, P., & Klinedinst, N. (2011). Introduction: Vagueness and language use. In Vagueness and language use (pp. 1-21). London: Palgrave Macmillan UK.

We agree that the relation between indirectness and vagueness has been sketched out a bit impressionistically in our original manuscript. We differentiate between vagueness of subjective predicates that constitute our empirical domain as a property of words and pragmatic ambiguity—a property of utterances when their meaning changes depending on discourse. We address this distinction in the *Background* section.

5. L88: on a possible loss of face, it seems to me that this is not only relevant for the speaker but also for the addressee of the comment, who might be offended by do opinion conveyed. So, both the speaker and the addressee would be subject to possible face loss in the situation that you envisage.

   Indeed, both the consideration of speaker's and listener's face may affect speaker choices. We now relate this discussion to previous literature and mention the Polyanna effect, where speaker's own face makes her favor positive utterances, as well as a number of politeness analyses that predict speaker's choice of indirect utterances as an attempt to provide positive feedback to the listener.

6. L96: regarding the coefficients in the equation, it would help if you could add an example with a specific value for the w.

   We now discuss how the weights of the w parameter translate into communicative behavior. A high value of w favors straightforward informative utterances, while a lower w allocates utility towards the social component, prioritizing conflict avoidance.

7. L224: about the politeness scenario, it is unclear to me why you are assuming that pretty good, including a rising intonation (indicated by the exclamation mark, I suppose) would be a case of indirect communication. The fact that this is a positive assessment is not at all ambiguous; therefore, it would not qualify as an indirect speech act.

   We agree that the example is not straightforward. We now replace the predicate "pretty good" with "not bad". Negated statements are often discussed in the politeness literature as being indirect.

8. L243: "Which is higher IN the direct utterance". You could also explain more clearly that amazing is compatible with fewer opinion statements compared to interesting, which means that amazing is more informative than interesting. (If I understood this correctly.)

   We have adopted this suggestion and clarify in the section *RSA models of social meaning* how informativity is calculated for these predicates. In the RSA-framework, to calculate informativity, we first need to fix the speaker's intended message. In the example, we assume that the speaker's opinion is strongly positive. We then show that the predicate "amazing" is more informative than "interesting" since "amazing" is more likely to lead the listener to recover the speaker's opinion.

9. L272-287: it makes sense to conceptualise agreement as a graded concept, but it would be helpful to illustrate the formalization using a concrete, real life, example.

   We have added an illustration of how utterance polarity and opinion certainty map the the values of a Beta-distribution to the section Opinions and their degree of alignment. Parameter β_1 controls the placement of the opinion on the negative-positive scale with low values of β_1 marking negative opinions. Parameter β_2 characterizes uncertainty: larger values of β_2 map to high opinionatedness. For example, a combination of low β_1 and high β_2 would correspond to a negative opinion and high certainty in that opinion. Formally, this will translate into

a narrow tall curve centered around negative values. A lower β_2 would indicate a lower degree of opinionatedness. A combination of a low β_1 and low β_2 would still map to a negative opinion but in that case the speaker is not very certain about the opinion being negative; a Beta-distribution in this case is still centered around negative values but it is wider and flatter indicating that the speaker's opinion might also include strongly negative and neutral values. To illustrate the effect of β-parameters on the distribution we have created a dynamic Shiny web-application, where users can manipulate the parameters and observe their effect on the shape of the distribution. The application is available at `https://cognitivemodeling.shinyapps.io/Indirectness/`

10. L340: What does "multi-objective" mean?

    We have clarified the sentence: we now say that the speaker chooses an utterance by taking into account multiple conversation goals.

11. L398-401: this should rather be part of the general discussion or the conclusion of the paper.

    We agree and have moved the considerations about the effect of utterance on opinion update to the *Discussion* section.

12. L418-425: at some point, you consider the possibility that the goal of communicating an opinion indirectly with a mitigated statement is a way to check speaker-recipient opinion alignment, which could be considered as a speaker-oriented goal. You also explain that you will not include this assumption in your model, but maybe you could add some clarification in the paper about which additional assumptions could (or could not) be incorporated in future elaborations of the model.

    Thank you for the chance to spell out some of the ways in which we see this analysis developing in the future! We hypothesize that the assumptions about relevant utility components will have the largest effect on model predictions. In the *Discussion* section, we now mention that the addition of opinion alignment goals is one of the possible paths to develop the current model. We further suggest that making social-utility models more general is a promising path to take, since most current models including ours only deal with a rather narrow set of communicative scenarios.

13. In Table 1, it is unclear what corresponds to A's beliefs and what corresponds to A's utterances.

    We have now specified in the Table 1 captions that the sentences in italics mark utterances, while labels in regular font mark opinions.

14. L464-5: about the typical cognitive limitation in everyday conversation, please add relevant references.

    In the *Summary: Modeling section*, we now cite several studies on iterated reasoning in strategic games that reveal the cognitive costs associated with employing higher-order reasoning. We further mention relevant work from the study of reference and comprehension of sentences with nested propositional-attitude verbs.

15. L484 on ethics: could you add the reference of the application and also how much money the participants received for taking part in the different studies. For the three experiments reported in the paper, it is necessary to include social demographic information about the sample of the population who participated in the studies, such as age (e.g., median, range) and gender in particular.

Ethics applications at the University of Tübingen are not associated with reference numbers but we now specify in the Behavioral data section that the decision letter was issued on 05.03.2021; the decision letter has been previously uploaded the the PLOS One submission system together with the manuscript. We now further provide the information about age and gender of our participants, as well as their hourly compensation rate (10 British pounds).

16. I am concerned that no information is provided about the sample size calculation (especially as the number of Ps for each experiment is different), the language of the stimuli, how the stimuli were selected, for instance, why these 10 topics, and whether or not the stimulate were presented in the random order.

    We have now clarified that the language of the stimuli is English and our participants all speak US-English as their mother tongue. We have further specified in the description of Experiment 1 that that we selected 10 subjective predicates that cover a range from strongly negative to strongly positive ones, taking inspiration from the choices reported in Yoon et al. (2020). As topics, we selected those that are likely to give rise to different opinions. We randomized the order of experimental stimuli.

    The number of participants in each experiment is indeed different, owing to differences in the experimental design. For Experiment 1, we were able to obtain multiple ratings for each predicate within participants. This design choice allowed us to settle for a lower number of participants (51). For other experiments, we were only able to present a subset of conditions to each participant. For example, in Experiment 3, we made sure that predicates in the first speaker's statement do not repeat within the experiment for a given participant, and that the second speaker's utterance is never the same as the first speaker's utterance. The latter requirement ensured that the dialogues sound more natural. Overall, Experiment 3 featured 32 conditions, and each participant was asked to evaluate 6 dialogues; it means that given randomization, we need data from approximately 6 participants to get 1 full set of data points. A sample size of 286 participants (274 in the analysis after exclusions) thus allowed us to collect approximately 51 data points per condition (we refer to them as design cells in the text). For each of the experiments, we have added a description of the experiment structure and number of observations per design cell. We hope that they make our choices more transparent.

17. L527: word semantics, do you mean "sentence semantics" or "compositional meaning"? As I wrote earlier, it is not totally clear to me that indirectness is a notion that applies to lexical items (unlike vagueness). This is an issue that should be clarified in the manuscript.

    We rephrased the sentence to clarify that by word semantics we mean a property of individual words, contrasting it with properties of utterances in discourse. We hope that our discussion of vagueness, ambiguity, and indirectness in the *Background* section provides further context for this distinction.

18. L538 and below: data WERE excluded (research data is a plural noun).

    Corrected.

19. L545: How is it possible not to have an informational goal when making an assertion? This should be clarified. I can think of examples, such as stating the obvious (also in indirect requests: "It's hot in here", meaning "Please open the window"), but it seems that you have something different in mind, here.

Our initial phrasing was indeed unclear. We imagined a speaker who does not intend to communicate their opinion but simply wants to avoid conflict. In that case, they may choose a statement that does not match their actual opinion but rather a statement that is closest to the listener's opinion. However, your skepticism finds support in our empirical data: speakers were not able to abandon informational goals in the social goals condition. The influence of informational goals in the "social-goals-only" condition manifests itself in the lack of substantial differences between the "social-goals-only" and "social-and-informational-goals" conditions. We have now added a clarification of what we mean by informational and social goals to the description of Experiment 2, and we discuss how communicative behavior would look like if speakers optimized only one of those goals.

20. L547: why did you only use very positive and very negative opinions for the experimental stimuli? This decision should be motivated.

    We now clarify our choices in section *Experiment 2: Pragmatic speaker*. For Experiment 2, we have chosen extreme ends of the opinion scale since they would potentially lead to greater differences in utterance choice when we manipulate communicative goals. If the speaker's opinion were neutral, any utterance would not result in a conflict (in the sense we operationalize it). Conflict is potentially maximal if the speaker's opinion is either strongly positive or strongly negative. We can then monitor whether the presence of social goals and the divergence between speaker's and listener's opinion affect utterance choice.

21. The scale on Figure 7 is ambiguous, as five hearts could also mean strongly negative. It will be important to add more information about the instructions that were given to the participants, specifically about how they were supposed to interpret the scale.

    We have added information about the instructions participants received to the description of Experiment 2. Participants were informed about the values of the scale during the instructions phase, we now mention it in the text. Additionally, every trial showed labels "strongly negative" and "strongly positive" that appeared to the left and to the right of the hearts accordingly. We understand that despite these clarifications participants could have interpreted the labels differently. For example, when participants rate women's access to reproductive care with five hearts, it could either mean that women already have excellent access to reproductive care or that they find it very important that women have access to reproductive care. This divergence of interpretations might be more likely for some topics than for others. However, our analysis of topic differences did not reveal any substantial effects in the evaluation of predicates.

22. L641: It is unclear what "a score" means, in the sense that it is difficult to understand the diagram. In other words more information about the dependent variable should be included.

    We agree that introducing a new term when reporting the results is not an optimal decision. We meant the value in hearts, we now make sure that we use this term consistently.

23. L701-2: Again, the theoretical background pertaining to the distinction between vagueness and indirectness should be improved to strengthen the interdisciplinary impact of this important piece of research.

    We have worked on clarifying this distinction throughout the text.

1 Reviewer 2

1. The manuscript by Dr. Asya Achimova and colleagues proposes an extension of the Rational Speech Act framework by incorporating social value functions to model the computational processes underlying conversational exchanges. The study primarily investigates simplified two-utterance interactions. Although the computational framework is elegantly formulated for this restricted setting, several key issues related to the treatment of communication context and underlying assumptions need to be addressed to bolster the study's generality and applicability.

   We are delighted to see that Reviewer 2 found our formalization elegant! We strove to address more substantial concerns in the revised manuscript.

2. Contextual Articulation of Social Value. The manuscript would benefit from a clearer articulation of the specific communicative contexts in which the proposed formulation of social value is most applicable. For example, in today's politically polarized climate—where listener opinions can be strongly divided and direct, unambiguous statements might be less favored—it is important to explain the relevance and limitations of the current formulation. A discussion of how these polarized contexts, which may be particularly prevalent in certain Western societies, influence the model's assumptions would strengthen the justification for the proposed utility function.

   We are grateful for the suggestion to relate our work to literature on political polarization. We now emphasize in the *Discussion* section that indirect communication allows conversation partners to approach sensitive topics and thus engage in dialogue.

3. Dimensionality of Communication Space. The assumption of a one-dimensional communication space in the context of polarized discourse requires further justification. The authors should elaborate on why and how the inherently multidimensional nature of real-world communication can be effectively reduced to a single dimension in this model. Providing theoretical or empirical support for this design choice is essential for establishing the generality of the framework.

   We would like to thank the reviewer for the opportunity to discuss to which extent our choice of representations affects the performance of the model! We fully agree that reducing the opinion space to one or two dimensions is a simplification of the actual communication process and of opinion representations. While representing opinions in full complexity is critical for some types of analysis, in our case, nothing in the model critically depends on the dimensionality of space. Our calculation of divergence can scale to higher-dimensional space, since calculating divergence between distributions is always possible, even though it can become mathematically more complex.

   We have added a more explicit discussion of dimensionality to the *Discussion* section. There we sketch out possible extensions of the model that could include more realistic opinion representations. We hope that our work will offer an architecture that will encourage refinement of individual model components.

4. Treatment of Ambiguity in Indirect Communication. The relationship between indirectness and ambiguity is nuanced. Indirect communication can stem from a compromise between expressing one's true intentions and preserving the interlocutor's face, potentially resulting in ambiguity that ranges from a narrow compromise to a multimodal distribution of meanings. The current use of a beta distribution may not fully capture these possibilities. It would

be helpful for the authors to test and discuss how their model accounts for various forms of ambiguity inherent in indirect speech.

It is indeed the case that indirect communication can stem from a variety of communicative goals on the one hand, and a variety of linguistic sources on the other hand. In this paper, we looked at subjective predicates and vagueness that is associated with them as a potential source of pragmatic ambiguity. We now provide a more explicit discussion of a relation between vagueness and indirectness in the *Background* section.

We agree that the choice of Beta-distributions, like any modeling choice, contains inherent limitations about the types of distributions we can represent. For example, representing a bimodal interpretation would indeed be difficult if not impossible. We see two arguments that make our choice justified for the purpose of current modeling. First, the shape of Beta-distributions closely matches the empirical distributions of utterance values that we obtained in Experiment 1. To explore whether choosing the empirical distribution from Experiment 1 vs. simulatd Beta-distributions affects model performance, we enabled the option to rely on the empirical distributions to calculate model predictions in the web-application that we developed for the model. It is avaliable at `https://cognitivemodeling.shinyapps.io/indirectness/`, the option to include empirical densities in the calculation is regulated by the switch "Use chosen densities for Utterance Choice and Opinion Inference" in the right panel of the main tab.

Second, the mechanism of taking into account the divergence between two distributions for utility calculation and inference is agnostic of the actual types of the distributions used. We discuss these arguments and offer directions for future elaborations of the model in the *Discussion* section.

5. Task Appropriateness for Socially Motivated Indirect Speech. There is a concern regarding whether the task employed in the study adequately captures the complexity of socially motivated indirect speech. For instance, expressions like "interesting" can convey dual meanings in indirect communication. The current experimental design may restrict the expression of such duality. Clarification on how the task allows for or limits the demonstration of these nuanced communicative intentions would be valuable.

   We are also concerned about the relative simplicity of our scenarios and the mode of presentation (reading) that may have obscured the meaning of predicates. Our goal in this work was to provide tasks that are maximally close to the settings under which our model operates. We wanted to ensure that the model and experiment participants have the same information about the conversation context, which forced us to be rather explicit in phrasing possible conversation goals, for example. We further decided to abstract away from utterance intonation as an important factor that could have affected the interpretation. The distribution of baseline utterance scores in Experiment 1 suggests that ironic interpretations were not very likely, and most participants interpreted predicates literally. At the same time, we cannot exclude that in Experiments 2 and 3 that provide more contexts, participants could have considered ironic interpretation. We now mention the limitations of our experimental paradigm in the *Discussion* section.

6. Informativeness versus Social Pay-off Trade-off. The manuscript posits a trade-off between informativeness and social pay-off. However, it is worth questioning whether this trade-off accurately reflects real-world communicative behavior. A more detailed discussion, potentially supported by empirical evidence, on how this relationship manifests in natural interactions

would help substantiate the model's assumptions.

We thank the reviewer for the opportunity to think about the actual communicative behavior in the wild and reach out to the field of conversation analysis. This literature indeed suggests that finding alignment and agreement in dialogue is important for human communication. We also find indirect evidence for the importance of social considerations in the literature on polarized discourse that shows that people prefer to avoid mentioning some topics entirely in light of social considerations. Furthermore, we see the politeness literature as illustrating the balancing of informational and social goals. We now mention these links more explicitly in the *Background* section.

Our data from Experiment 2 further suggest that speakers do strike a balance between informativeness and conflict avoidance. We agree that our choice of utterances might have made this effect more pronounced. In natural communication, if there are utterances that allow the speaker to remain maximally informative and at the same time avoid possible conflict even when the opinions do not align, such a trade-off would not be necessary. We return to this issue in the *Discussion* section and spell out how having a less scripted conversation might enable speakers to find ways to satisfy both goals without compromising either of them. It is further possible, that at the individual level, speakers do not show this trade-off as obviously. However, since we engage in population-level modeling at this stage, modeling it as a trade-off appears most fruitful. Once more, we see this model as a first step into conceptual analysis to further refine downstream empirical questions for more ecologically valid data sources as well.

7. Given that the alignment with the listener's opinion appears to be a key motivational factor in the model, it is surprising that this motivation is not manipulated within the simulations. While Experiment 2 is consistent with the experimental design, it would be instructive to see how this manipulation might influence outcomes in a subsequent experiment (e.g., Experiment 3). The authors should discuss potential simulation results and their implications for the model's predictive power.

   We appreciated this suggestion and added a model-simulation where the social goals are set to 0 to the section *Experiment 3: Pragmatic listener*. We discuss that when the model ignores social goals of the speaker, it interprets the second speaker's response at face value: the lines that represent the inferred opinion are then flat since they do not depend on the first speaker's utterance anymore. We have added a figure (S5 Fig) to the *Supplementary information* section and made it possible to flexibly manipulate several model parameters and observe the effect of these adjustments on model predictions in the R Shiny web-application that accompanies the paper. It is available at `https://cognitivemodeling.shinyapps.io/Indirectness/`. The application link is now included in the manuscript.

   While modeling allows us to explore what happens when social goals are set to 0, we believe it might be not possible to induce exactly the same effect in a behavioral experiment. As results of Experiment 2 show, speakers are reluctant to abandon information goals entirely even when explicitly instructed to do so: they still pick utterances that to a certain extent reflect the true opinion of the speaker even when we say that the communicative goal is just to avoid conflict. We take an opportunity to mention this important benefit of modeling in the *Discussion* section: modeling allows us to spell out and examine simulations that might be unattainable in behavioral studies due to a variety of constraints. In doing so, we can examine the consequences of our modeling choices and the implications they carry for the model predictions.

8. Clarity of Expression in Examples. The example labeled "5-a" appears to be an overly indirect or ambiguous expression of negative opinion. Consider revising this example to include a more direct expression, which would help clarify the intended communicative intent.

   We share this concern, so we decided to add clear labels that show whether the utterances signal a positive or a negative consequence. We have debated simplifying the examples, but we feared running into the risk of making our dialogues even more simplified and thus distant from actual communication. We hope that our solution with labeling the utterances provides a compromise between keeping more naturalistic examples and getting our idea across in a more straightforward way.

9. Quality of Figures. Figure 4 is currently too blurry to be clearly interpretable. Please provide a higher-resolution version to ensure that all details are visible.

   We have uploaded a higher resolution image for Figure 4.

10. Comparison with Politeness Models. The manuscript would benefit from a clearer comparison with existing politeness models, especially those previously proposed by the authors. Highlighting the novel contributions of the current study relative to these models will clarify its significance.

    We now make a more explicit comparison to politeness models in the *Discussion* section. We mention that our model differs from others in several important respects. Politeness models have simplified social utility components where the utility of utterances is (a) fully known; and (b) is determined by the value to the listener. This form of utility often means that positive utterances carry higher utility than the negative ones. We show that politeness models therefore cannot capture the dependence of the utterance utility on the opinion of both conversation partners, and make an incorrect prediction that positive utterances should be preferred if social utility is to be maximized. The model we propose is able to make more nuanced predictions: it is not the utterance polarity that determines social utility but the divergence of opinions of conversation partners. Thus, AMIC predicts that both positive and negative utterances can score high on social utility depending on whether their choice can lead to conflict.

11. Data Presentation and Variability. The figures currently lack error bars, which are crucial for conveying the variability of responses across subjects. Please include error bars in the relevant figures to enhance the clarity and reliability of the data presentation.

    We have recreated the plots for Experiments 2 and 3 to include 95% confidence intervals.

12. Model Comparison and Alternative Explanations. The data presented appear to be interpretable by several different models. It would be helpful to discuss potential alternative models and to provide a comparison of their explanatory power relative to the proposed model.

    There are a number of ways in which we could see constructing alternative models. One of the ways is relying on a different method of calculating divergence. We mention Earth-Movers distance and Jensen-Shannon divergence and provide figures in the *Supplementary Information* section that illustrates how model predictions change when we rely on those divergence measures. We also enabled the option to choose a divergence measure in the R Shiny app.

    Another way in which models could differ is the architecture of the social utility. We here refer the reader to the discussion of the differences between politeness models and AMIC in

the *Discussion* section. Except for politeness models, which are not adequate to capture our utterance choice and inference data, we are not aware of other competitor models currently on the market. However, we sincerely hope that our work will spark new proposals that either offer modular extensions of our work or work out alternative formalizations for the processes that we model.

13. Figure Axis Legend in Figure 11. The y-axis legend in Figure 11 requires additional clarification. Please provide more detailed information regarding what the y-axis represents to ensure that the figure is self-explanatory.

    We have renamed the y-axis and provided a more detailed caption for the figure.
